# Prediction Medicine: Biomarkers, Risk Calculators and Magnetic Resonance Imaging as Risk Stratification Tools in Prostate Cancer Diagnosis

**DOI:** 10.3390/ijms20071637

**Published:** 2019-04-02

**Authors:** Daniël F. Osses, Monique J. Roobol, Ivo G. Schoots

**Affiliations:** 1 Department of Radiology & Nuclear Medicine, Erasmus University Medical Center, 3015 GD Rotterdam, The Netherlands; i.schoots@erasmusmc.nl; 2 Department of Urology, Erasmus University Medical Center, 3015 GD Rotterdam, The Netherlands; m.roobol@erasmusmc.nl

**Keywords:** prostate cancer detection, risk stratification, biomarker, risk calculator, magnetic resonance imaging, cost-effective diagnostic pathways

## Abstract

This review discusses the most recent evidence for currently available risk stratification tools in the detection of clinically significant prostate cancer (csPCa), and evaluates diagnostic strategies that combine these tools. Novel blood biomarkers, such as the Prostate Health Index (PHI) and 4Kscore, show similar ability to predict csPCa. Prostate cancer antigen 3 (PCA3) is a urinary biomarker that has inferior prediction of csPCa compared to PHI, but may be combined with other markers like TMPRSS2-ERG to improve its performance. Original risk calculators (RCs) have the advantage of incorporating easy to retrieve clinical variables and being freely accessible as a web tool/mobile application. RCs perform similarly well as most novel biomarkers. New promising risk models including novel (genetic) markers are the SelectMDx and Stockholm-3 model (S3M). Prostate magnetic resonance imaging (MRI) has evolved as an appealing tool in the diagnostic arsenal with even stratifying abilities, including in the initial biopsy setting. Merging biomarkers, RCs and MRI results in higher performances than their use as standalone tests. In the current era of prostate MRI, the way forward seems to be multivariable risk assessment based on blood and clinical parameters, potentially extended with information from urine samples, as a triaging test for the selection of candidates for MRI and biopsy.

## 1. Introduction

Although the European Randomized study of Screening for Prostate Cancer (ERSPC) and the recent analyses from the Prostate, Lung, Colorectal, and Ovarian (PLCO) Cancer Screening Trial show evidence that prostate-specific antigen (PSA)-based screening significantly reduces prostate cancer (PCa)-specific mortality, screening for PCa remains a controversial issue [1,2,3,4,5]. False positive PSA tests in patients with benign prostatic hyperplasia (BPH) and/or prostatitis result in unnecessary testing (performance of unnecessary systematic transrectal ultrasound [TRUS]-guided prostate biopsy [SBx]). In addition, PSA-based screening can lead to the overdiagnosis, and potentially overtreatment, of PCa which will never become clinically significant. These harms have a significant effect on the quality of life and therefore diminish the number of quality-adjusted life years (QALYs) due to PSA-based PCa screening. Refinements to the PCa diagnostic pathway, focusing on detecting only those cancers that are potentially life-threatening, are needed to make the pathway less burdensome to patients and as such more cost-effective and acceptable to the general population and health care providers [6].

International guidelines propose such refinements in men requesting their physician to “early detect” PCa by recommending an individualized opportunistic PCa screening policy [7,8]. This opportunistic screening goes along with shared informed decision-making, taking into account the individual potential advantages and damages related to PSA testing [7,8]. Furthermore, guidelines recommend the use of risk stratification tools, such as novel biomarkers, risk calculators (RCs) and magnetic resonance imaging (MRI), for the prediction of a positive prostate biopsy as reflex tests after an elevated PSA level [9,10,11,12,13,14,15,16,17]. This may support the process of shared informed decision-making, reduce the number of unnecessary biopsies by better identification of those men at risk of PCa, and better differentiate aggressive from non-aggressive cancers.

While these risk stratification tools have additional value within the diagnostic pathway, physicians should ask themselves if these tools are necessary in every man with an elevated PSA level, taking into account the height of its additional diagnostic and predictive information, the burden for the patient, the availability and costs for society. Risk stratification could be based on one tool. Performing additional tests only in those men considered to be at high-risk of having clinically significant PCa (csPCa) (defined as Gleason score [GS] ≥ 3 + 4 or ≥ International Society of Urological Pathology (ISUP) grading group 2) could be an acceptable option [18,19]. The risk of promising “easy-to-perform” tools is extensively (and unnecessary) testing all men (not only the high-risk men), which could result in the opposite effect than intended; to be specific and cost-effective [20]. Clear and explicit directions for diagnostic pathways that combine risk stratification tools after an elevated PSA level in order to potentially reduce the number of tests without missing csPCa are currently lacking.

The aim of this review is to discuss the most recent advancements of state-of-the-art risk stratification tools in the detection of csPCa, and their application in contemporary practice. Furthermore, we evaluated diagnostic pathways that combine several stratification tools to potentially realize a high csPCa detection rate together with a high cost-effectiveness.

## 2. Novel Biomarkers and Risk Calculators in Prostate Cancer Diagnosis

Several PSA derivatives have been proposed as PCa biomarkers to improve the specificity of the PSA test. The percentage of free PSA (fPSA) to total PSA (tPSA) was introduced three decades ago but this test improved clinical judgment only when levels reached extreme values [21]. More recently, fPSA has been found to include the isoforms benign PSA (bPSA), proPSA (with its most stable form [−2]proPSA) and intact PSA (iPSA) with usefulness in the detection of PCa [22]. Combining these isoforms has resulted in the Prostate Health Index (PHI) and four-kallikrein (4K) panel. Furthermore, molecular biology has allowed the study of genes associated with PCa. Next to novel biomarkers many RCs have been developed to predict biopsy outcome. In addition, novel biomarkers have been incorporated into existing RCs and new PCa risk models including novel biomarkers have been developed (e.g. SelectMDx, Stockholm-3 [S3M]) (Table 1). 

### 2.1. Blood-Based Biomarkers: Prostate Health Index and Four-Kallikrein Panel

The PHI test result is based on the following mathematical formula: ([–2]proPSA/fPSA x √PSA) and is developed to predict the probability of any PCa and csPCa at prostate biopsy. PHI is the least expensive ($80 in the USA) of currently available commercial multiplex biomarkers and is suggested in the initial and repeat biopsy settings [8,23,24]. On average, using the PHI with a cut-off of ≥25 to biopsy could avoid 40% of biopsies and reduce 25% of GS 6 diagnoses at the cost of missing 5% csPCa [25]. Recently, Chiu et al. compared the performance of PHI in different ethnic groups from nine sites (1688 Asian and 800 European men), concluding that PHI was more effective in safely reducing biopsies in Asian men compared to European men (56% versus 40% biopsy reduction) [26].

The 4Kscore is based on serum biomarkers (i.e. the 4K-panel = tPSA, fPSA, iPSA and human kallikrein 2 [hK2]) and includes clinical variables like age, digital rectal examination (DRE) and prior biopsy results to predict the risk of csPCa on biopsy. The 4Kscore is a commercially available assay, it is not available in Europe and costs around $500 in the USA [27]. Its use is recommended in patients undergoing initial and repeat biopsy [28]. A systematic review to evaluate the performance of the 4Kscore in the pre-biopsy setting showed a pooled area under the curve (AUC) above 0.80 for the discrimination of csPCa, which was highly consistent across 11 studies involving over 10,000 subjects [29]. The AUC of the receiver operating characteristic (ROC)-curve summarizes the value of a test. The higher the AUC of the ROC-curve is, the more combinations of high sensitivity and specificity are available, thus the better the test performs. On average, using the 4Kscore with a cut-off risk of 9% csPCa to indicate systematic biopsy (SBx) could avoid 43% biopsies at the cost of missing 2.4% csPCa [12,30,31]. In a comparative study including 531 men undergoing first-time biopsy, Nordström et al. found that the PHI test and 4Kscore showed similar ability to predict the detection of csPCa (AUC 0.71 versus 0.72) [32]. In summary, the serum-based biomarkers PHI and 4Kscore show comparable performance but are substantially different in price.

### 2.2. Urine-Based Biomarkers: PCA3, TMPRSS2-ERG, HOXC6, TDRD1 and DLX1 Genes

Prostate cancer antigen 3 (PCA3) is a gene that transcribes a long non-coding messenger RNA (mRNA) that is overexpressed in PCa tissue and is detectable in urine after DRE. The PCA3 score is calculated measuring the concentration of PCA3 mRNA in relation to PSA mRNA and costs around $300 in the USA [33]. Guidelines recommend using a cut-off of 35 in men with moderately elevated PSA for whom repeat biopsy is being considered [8,28]. Numerous studies indicate that the PCA3 score has greater accuracy for overall PCa detection in the repeat biopsy setting compared to tPSA and fPSA [34,35,36]. Data about the association of the PCA3 score with csPCa are, however, conflicting [37,38,39,40]. In recent years, comparative studies have demonstrated that PHI outperforms PCA3 for the prediction of csPCa on biopsy [41,42]. As the current paradigm emphasizes detection of csPCa, the potential of PCA3 as a reflex test is questionable.

Another gene associated with PCa and detectable in urine after DRE is TMPRSS2-ERG fusion. Studies demonstrated that the *TMPRSS2-ERG* fusion gene has a greater diagnostic accuracy than tPSA, with a high specificity (93%) and positive predictive value (PPV) (94%) for the detection of PCa [43,44]. Unlike PCA3, TMPRSS2-ERG levels were associated with csPCa. However, its low sensitivity reduces its value as a standalone test. Combining PCA3 with TMPRSS2-ERG can improve the prediction of csPCa [15,43,44]. A commercial test, the MiProstate Score (MiPS), incorporates PSA, PCA3 and TMPRSS2-ERG to predict the risk of PCa and csPCa. MiPS costs around $700 in the USA and is a promising test following PSA screening, but has not yet been validated in prospective studies and directly compared with other biomarkers [45,46].

Microarray analysis of mRNA from PCa tissue compared with normal prostate tissue revealed 39 potential biomarker candidates [40]. Among them, eight mRNAs were upregulated in precipitates of urine obtained after DRE from men with PCa. From these eight genes a panel (*HOXC6*, *TDRD1* and *DLX1*) was selected for the detection of PCa and in particular csPCa [47,48]. This urinary three-gene panel showed higher accuracy (AUC 0.77) to predict csPCa in biopsies compared with the PCA3 score or serum PSA.

### 2.3. Combinations of Biomarkers and Clinical Data = Risk Calculators

#### 2.3.1. Risk Calculators Including Only Standard Clinical Parameters

RCs have the advantage of incorporating easy to retrieve clinical variables. A systematic review identified 127 existing RCs in the field of PCa [9]. Only six RCs to predict biopsy outcome have been externally validated in more than five study populations other than the development population: the ERSPC Rotterdam Prostate Cancer Risk Calculator (RPCRC), the Finne model, the Chun model, the Karakiewicz model, the Prostate Cancer Prevention Trial (PCPT) model and the ProstataClass model [10,49,50,51,52,53]. Besides PSA, the DRE was the most common predictor variable to be included in the risk models, followed by age, fPSA and transrectal ultrasound (TRUS) prostate volume (PV). In a recent head-to-head comparison, RCs incorporating PV were shown to be superior in identifying men at risk of csPCa [54]. Therefore, the incorporation of PV into RCs is recommended [54,55,56]. The same study showed that the above-mentioned RCs and the so-called Sunnybrook RC have a moderate to well discriminatory ability when predicting any PCa (AUCs from 0.64 to 0.72) [54,57]. The ERSPC RPCRC was shown to be slightly superior in predicting men at risk of csPCa. On average, using the ERSPC RPCRC with biopsy at a cut-off of 4% csPCa risk could avoid 32% of biopsies and reduce 25% of GS 6 diagnoses while keeping a 95% sensitivity for detecting csPCa [54].

Another advantage of RCs using only readily available clinical data is that they are available as web tool and mobile applications (Apps), making (most of) them freely-accessible for everyone [58]. A recent systematic review assessing the everyday functionality and utility of the currently available RC Apps showed that based on the Mobile Application Rating Scale, the ERSPC RPCRC App performed well [59].

#### 2.3.2. Risk Calculators Including Novel Biomarkers next to Clinical Parameters

The original RCs were virtually all developed in the 1990s. That means that they do not include later-developed biomarkers. The addition of PHI to the ERSPC RPCRC 3 (initial biopsy) and four (repeat biopsy) significantly improved the prediction of csPCa [60,61]. More recently, Loeb et al. confirmed the added value of PHI when incorporated into the PCPT RC and ERSPC RPCRC, and created a new PHI-based prediction model with an AUC of 0.75 [62].

The 4Kscore is in fact a risk prediction model combining novel biomarkers (i.e. the 4K-panel) and standard clinical data. Verbeek et al. recently investigated in a cohort of 2872 men (initial biopsy) the clinical impact of the 4Kscore, ERSPC RPCRC and the combination of both for predicting csPCa [63]. In this study the 4Kscore and ERSPC RPCRC had similar AUCs (0.88 versus 0.87). The 4Kscore-ERSPC RPCRC combination significantly improved the AUC to 0.89 [64]. Gains in net benefit must, however, be weighed against additional costs and the availability of tests.

The PCA3 score has also been investigated in conjunction with other variables. Hansen et al. designed a PCA3-based nomogram specifically to predict initial prostate biopsy results [65]. This model could lead to the avoidance of 55% biopsies while missing 2% of patients with csPCa. PCA3 has also been incorporated into existing prediction tools for men undergoing initial or repeat biopsy, such as the ERSPC RPCRC, PCPT RC (updated in 2018 with TMPRSS2-ERG added) and Chun model [66,67,68,69,70]. Incorporation of PCA3 improved the diagnostic accuracy of all RCs, which is perhaps the most appropriate application of PCA3 [71]. Similarly, the addition of MiPS to the PCPT RC was superior to a base model [46]. Using various cut-offs, the MiPS-PCPT RC model would avoid 35–47% of biopsies while missing 6–10% low-risk PCa and 1.0–2.3% csPCa.

Based on the high predictive accuracy for csPCa of the urinary three-gene panel—*HOXC6*, *TDRD1* and *DLX1*—Van Neste et al. developed a new risk model combining *HOXC6* and *DLX1* with clinical parameters (age, PSA, DRE, PV and family history). This model is available as the SelectMDx test and costs around €300 in Europe [40,72]. The European Association of Urology (EAU) guidelines suggest considering the use of SelectMDx in deciding whether to take an initial or repeat biopsy [8]. The model demonstrated an AUC of 0.86 for csPCa and outperformed the base model without mRNA markers and the PCPT RC. Decision curve analysis suggested that SelectMDx could reduce 42% of biopsies while missing 2% csPCa. Recently, analyses showed that with SelectMDx quality-adjusted life years (QALYs) could be gained while saving healthcare costs in the initial diagnosis of PCa, making the use of SelectMDx before proceeding to biopsy potentially a cost-effective strategy [73,74,75]. As stated by Van Neste et al. the SelectMDx model is mainly driven by the strong predictive value of PSA-density [72].

Another new risk model is the S3M. This model is based on plasma protein biomarkers (PSA, fPSA, iPSA, hK2, MSMB, MIC1) combined with genetic polymorphisms (232 single nucleotide polymorphisms) and clinical variables (age, DRE, PV, family and biopsy history). The model was created using data from the Stockholm-3 study, with PSA-density being once more the strongest predictor [76]. The S3M is not available outside of Sweden and it is difficult to judge its exact price [77]. The S3M is proposed to be used in the initial biopsy setting. In a screening cohort, the S3M performed significantly better than PSA alone for the detection of csPCa (AUC 0.74 versus 0.56) [76]. At the same level of sensitivity as the PSA test using a cut-off of ≥3.0 ng/mL to diagnose csPCa, use of the S3M could reduce the number of biopsies by 32% and avoid 17% GS 6 diagnoses [78]. Recently, the S3M was updated and showed a slightly improved AUC [77]. In a contemporary independent cohort, the S3M also performed well (38% biopsy avoidance at the cost of missing 6% csPCa) [79]. The S3M’s performance characteristics should be compared with other biomarkers and RCs before wide incorporation in daily practice.

## 3. Magnetic Resonance Imaging (MRI) as Clinical “Biomarker” in Prostate Cancer Diagnosis

With the technological advancements in recent years and increasing experience among technicians, radiologists, urologists and pathologists, MRI has evolved as an appealing tool in the diagnostic arsenal [8]. MRI has shown to be the preferred imaging modality for detecting areas suspicious for csPCa and allowing guidance for targeted biopsy (TBx), with a total cost of $700–$3000 depending on regional differences in healthcare systems outside of Europe [80,81]. In Europe, the costs of a prostate MRI is estimated to be €300–€500 [81]. TBx can be performed using in-bore MR-guided biopsy, cognitive fusion biopsy and software fusion biopsy, without significant differences in the detection rate of csPCa among the three techniques [82]. TBx is most often performed in combination with SBx. Guidelines for standardized prostate MR image acquisition and reporting are published [83]. The Prostate Imaging—Reporting and Data System (PI-RADS) describes the assessment of MRI lesions, judged on a likelihood scale from 1 to 5. A PI-RADS assessment score of 3 to 5 is mostly used as definition for a suspected lesion on MRI [83]. Strategies incorporating MRI as a (subjective) ‘biomarker’ in different clinical settings have been undergoing investigation or are still being investigated. In addition, to better identify those men who would benefit from TBx and/or additional SBx after an MRI scan, MRI data have been combined with (objective) novel biomarkers and incorporated into existing and new developed risk models (Table 1).

### 3.1. Initial Biopsy Setting

Although MRI with or without TBx (MRI strategy), in addition to or as a replacement of SBx, is increasingly investigated in the initial biopsy setting, guidelines do not yet recommend a pre-biopsy MRI or an upfront MRI-directed biopsy management in biopsy-naïve men [8,28]. Over the last years, studies have shown that MRI in combination with TBx significantly improved the detection rate of csPCa in the repeat biopsy setting but not (yet) in biopsy-naïve men [80,84]. High-level evidence for csPCa detection by the MRI strategy as compared to SBx in biopsy-naïve men has been scarce until 2018.

Recently, two multicenter randomized controlled trials (RCTs) in biops- naïve men investigated the performance of the MRI strategy versus SBx [17,85]. The PRECISION trial showed that MRI in combination with TBx detected 12% more csPCa and 13% less low-risk PCa (=GS 6 PCa or ISUP grading group 1) than SBx, while a 28% reduction of biopsies was realized. Porpiglia et al. also concluded that the MRI strategy outperformed SBx. Furthermore, two prospective multicenter studies investigating the agreement of PCa detection between the MRI strategy (i.e. without additional SBx) and SBx in biopsy-naïve men have been published recently [86,87]. In the 4M-study and MRI-FIRST trial the proportion of detected csPCa by MRI with or without TBx (25%–32%) was similar to the proportion csPCa detected by SBx (23%–30%). However, the MRI strategy detected significantly less low-risk PCa compared to SBx and MRI could have avoided 18%–49% of biopsy procedures at the cost of missing 5% csPCa. Lastly, a Cochrane review determined in a mixed biopsy population (initial and repeat) that at a prevalence of 30% csPCa, the negative predictive value (NPV) for MRI, MRI-TBx, MRI strategy and SBx was 90%, 93%, 90% and 87% (using template biopsy as reference standard), respectively [88]. An additional agreement analysis showed an equivalent proportion of detected csPCa by MRI with or without TBx (22%) and SBx (20%) in biopsy-naïve men. However, the MRI strategy beneficially avoided the detection of a significant proportion (37%) of low-risk PCa and reduced 32% of biopsy procedures (negative MRI) at the cost of missing 4% csPCa, across 20 included studies involving over 5000 biopsy-naïve subjects.

### 3.2. Repeat Biopsy Setting

Guidelines recommend the use of MRI and TBx in the setting of persistent clinical suspicion of PCa after previous negative SBx [8,28,89]. Studies have shown that the MRI strategy can significantly improve the detection of csPCa while reducing the detection of low-risk PCa and number of performed biopsy procedures in comparison to repeat SBx [80,84,90,91,92,93]. The NPV of the MRI strategy in this setting is, however, also not 100% [94,95,96].

The Cochrane review from Drost et al. included 10 studies involving over 1500 subjects to determine the agreement of PCa detection between the MRI strategy and SBx in the repeat biopsy setting [88]. The analysis showed that the MRI strategy detected 44% more csPCa than SBx. Furthermore, the MRI strategy avoided the detection of a significant proportion (38%) of low-risk PCa and reduced 32% of biopsies at the cost of missing 2% csPCa.

### 3.3. Novel Biomarkers and MRI Merged Together

Gnanapragasam et al. showed in 279 men requiring a repeat biopsy that adding PHI to the MRI suspicion score improved csPCa prediction (AUC 0.75) compared to PSA + MRI alone (AUC 0.69). Using a PHI cut-off ≥35, 13% of low-risk PCa and 5% of csPCa was missed while 42% of men potentially spared a repeat biopsy [97]. Recently, Druskin et al. showed in men with previous negative biopsy that PHI-density and PI-RADS score were complementary, with a PI-RADS score ≥3 or, if PI-RADS score ≤2, a PHI-density ≥0.44, being 100% sensitive for csPCa. Using 0.44 as a threshold for PHI-density combined with MRI, 35% of biopsies could have been avoided at the cost of missing 8% csPCa [98].

In a population of 300 men (initial and repeat biopsy) the combined use of 4K and prostate MRI showed to be superior in the prediction of csPCa (AUC 0.82) and patient’s selection for biopsy, compared to using the 4Kscore (AUC 0.70) or PI-RADS score (AUC 0.74) individually [99]. If one was to defer a biopsy in men with a negative MRI and a 4Kscore <7.5%, one would avoid 15% of the biopsies and miss 2% csPCa.

### 3.4. Risk Calculators Including MRI Data

Kim et al. determined the added value of MRI to the PCPT RC in the detection of csPCa on TBx and/or SBx in 339 men requiring initial or repeat biopsy [100]. In patients with an estimated risk of csPCa ≤10%, the use of MRI in addition to the PCPT RC provided a significant improvement in clinical risk discrimination (AUC 0.60 versus 0.69). Radtke et al. added pre-biopsy MRI data (PI-RADS v1 score) to the ERSPC RPCRC parameters and developed newly fitted RCs that were superior to ERSPC RPCRC and PI-RADS score alone in their study cohort [101]. However, net benefit of these RCs was observed only beyond the 10% risk threshold for csPCa. Recently, Alberts et al. improved the ERSPC RPCRCs. They used a multicenter cohort of 961 men who underwent SBx with or without TBx, and added next to PI-RADS v1 score age as parameter to the ERSPC RPCRCs [102]. For the MRI-ERSPC RPCRC 3 net benefit was only observed above a 10% risk threshold for csPCa, which would result in 14% biopsies avoided while missing low-risk PCa in 13% and csPCa in 10% of biopsy-naïve men. The MRI-ERSPC RPCRC 4 would have avoided 36% of repeat biopsies, missing low-risk PCa in 15% and csPCa in 4% of men.

Other groups developed new MRI-based prediction models. Van Leeuwen et al. constructed a model based on the data of 393 biopsy-naïve men undergoing template biopsy with or without TBx incorporating the same parameters as used in the MRI-ERSPC RPCRCs. Using a csPCa risk threshold of 10% would have avoided 28% of biopsies in their cohort, missing 13% low-risk PCa and 3% csPCa [103]. Truong et al. developed a nomogram for predicting benign pathology on TBx in the setting of an abnormal MRI after previous negative biopsy [104,105]. The model (PSA, age, PV and PI-RADS v2 score) had an AUC ranging from 0.77 to 0.80. At a benign pathology risk threshold of 70% to biopsy, 29% of biopsies could be avoided with 14% low-risk PCa and 8% csPCa being missed. Recently, Mehralivand et al. constructed a RC to differentiate among patients with positive MRI findings who would benefit from TBx and SBx from those who would not [106]. At a csPCa risk threshold of 20% to biopsy, 38% of biopsies could have been avoided while identifying 89% of csPCa.

Again, we are close to being confronted with dozens of RCs predicting biopsy outcome using amongst others MRI results. To avoid this, it is strongly advised that the publication of yet another model should only be pursued after performance is compared with already available models that have shown good discriminative capability. Calibration to a particular setting is relatively easy to do (provided that the predictive effects of other covariates are similar between the development and designated clinical setting), as now is stated in the new MRI-ERSPC RPCRC App. In that way we will create a situation where the best-performing model (both with respect to discrimination and calibration) will be used and that results can be compared that may potentially lead to further refinement.

## 4. Diagnostic Pathways that Combine Risk Stratification Tools in Prostate Cancer Diagnosis

**Figure 1:**
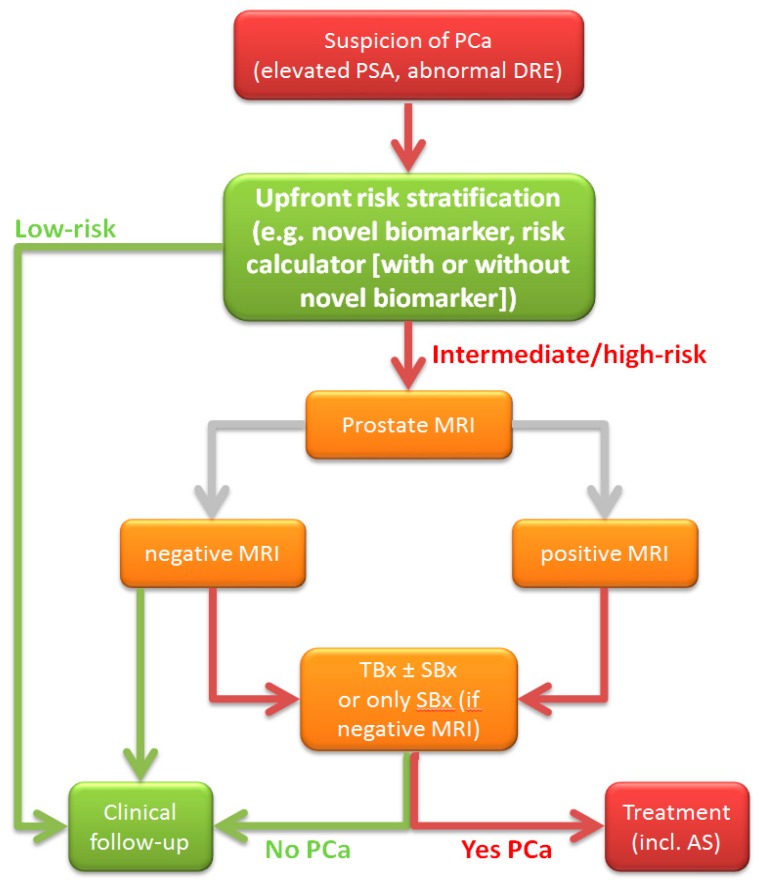
Flowchart of men with elevated prostate-specific antigen (PSA) and/or abnormal digital rectal examination (DRE), with the combination of upfront risk stratification and if indicated prostate MRI and biopsy. PSA: prostate-specific antigen; DRE: digital rectal examination; PCa: prostate cancer; MRI: magnetic resonance imaging; PI-RADS: MRI suspicion score; TBx: MRI-targeted biopsy; SBx: systematic biopsy; AS: active surveillance.

Prostate MRI seems to be the most useful risk stratification tool because of its ability to detect suspicious lesions and guide for TBx, next to inform one about the risk at csPCa (PI-RADS score). However, in a considerable proportion of patients the MRI will not show any abnormalities making it thereby potentially a redundant test. In addition, some patients will have false positive abnormalities on MRI (i.e. benign pathology or low-risk PCa) resulting in unnecessary TBx. The state-of-the-art challenge in the current MRI era is to identify those men who will benefit from an MRI with TBx, for maximum csPCa detection while reducing the number of unnecessary MRIs, biopsies and diagnoses of low-risk PCa. An option could be upfront risk stratification with a novel biomarker or RC (with or without novel biomarker(s) included), and if indicated subsequent MRI with if indicated subsequent biopsy (Table 1 and Figure 1).

### 4.1. Upfront Novel Biomarker and If Indicated Subsequent MRI and Biopsy

PHI has been tested as a predictor of a positive MRI in men requiring repeat biopsy [97]. PHI scores were generally higher in men with an MRI lesion. However, using PHI only marginally increased predictive value compared to PSA in this study suggesting that PHI is unlikely to be useful as a triaging test in deciding if an MRI will be positive. Punnen et al. looked at different sequencing strategies to combine the 4Kscore and MRI in a mixed biopsy population (initial and repeat) [99]. A strategy of doing an initial 4Kscore, followed by an MRI if the 4Kscore was greater than 7.5% and a subsequent TBx if the MRI was positive showed a 25%, 83% and 75% reduction in the number of MRIs, biopsies and low-risk PCa diagnoses, respectively. However, this strategy resulted in 33% of csPCa being missed. A similar pathway using PCA3 score ≥35 as threshold would result in 52% MRI reduction, 76.4% reduction of biopsies and 86.6% less diagnoses of low-risk PCa, at the cost of missing 47.5% csPCa [107]. All studies conclude that optimized sequencing of novel biomarkers and MRI is the other way around, i.e. an initial MRI followed by a novel biomarker among only those men with a low to moderate suspicion score on MRI. However, that still would mean at least an MRI in every man with a suspicion of PCa.

### 4.2. Upfront Risk Calculator Including only Standard Clinical Parameters and If Indicated Subsequent MRI and Biopsy

Alberts et al. studied whether upfront risk stratification with the ERSPC RPCRC could be used before the decision to perform an MRI in men confronted with a previous negative SBx while having a persistent suspicion of csPCa [19]. The analysis was restricted to TBx outcomes. In their cohort, upfront ERSPC RPCRC-based patient selection for MRI would have avoided 51% of MRIs, 69% of biopsies and 25% of low-risk PCa diagnoses, while missing 10% csPCa. In a repeat biopsy setting Drost et al. found that upfront use of the ERSPC RPCRC to select men for MRI with TBx could diagnose most of the csPCa (83%), while saving 37% of MRIs, 55% of biopsies and 66% of low-risk PCa diagnoses [108].

Recently, Mannaerts et al. showed in a retrospective biopsy-naïve cohort of 200 men that a pathway of initial ERSPC RPCRC, followed by an MRI if the ERSPC RPCRC advised to perform biopsy and subsequent SBx with additional TBx in case of a positive MRI, would reduce 37% of MRIs and biopsies, 23% of low-risk PCa diagnoses while missing 6% csPCa [109]. A TBx-only strategy after ERSPC RPCRC would have missed 27% of csPCa in this cohort. Currently, a Dutch prospective study (MR PROPER) evaluating the MRI strategy versus SBx in biopsy naïve men (3000 inclusions aimed), both after initial risk stratification with the ERSPC RPCRC, is ongoing and will provide more clarity about the value of the ERSPC RPCRC-MRI pathway [110]. In any case, results obtained till now argue for an ERSPC RPCRC-based selection for MRI with performance of only MRI with or without TBx in repeat biopsy men considered to be at high-risk of csPCa according to the ERSPC RPCRC, while biopsy-naïve men considered to be at high-risk should undergo both MRI with or without TBx and SBx.

### 4.3. Upfront Risk Calculator Including Novel Biomarker(s) and If Indicated Subsequent MRI and Biopsy

In a retrospective study the SelectMDx score was significantly higher in patients with a suspicious lesion on MRI compared to patients with a negative MRI. For the prediction of MRI outcome, the AUC of SelectMDx was 0.83 compared to 0.66 for PSA and 0.65 for PCA3, suggesting a positive association between SelectMDx and the final PI-RADS v2 score [111]. Trooskens et al. presented data on the use of SelectMDx (including TRUS PV) to exclude low-risk patients from undergoing an MRI [112]. A strategy of doing upfront SelectMDx, followed by an MRI if the risk for csPCa was greater than 10% and subsequent SBx with additional TBx if the MRI was positive (defined as PI-RADS score ≥4), would reduce 35% of MRIs and biopsies, 52% of low-risk PCa diagnoses while missing 2% csPCa.

Grönberg et al. recently investigated the combination of S3M and MRI in a cohort of 532 men who were referred for PCa workup (initial and repeat biopsy). Performing MRI with or without TBx and additional SBx only in men with a risk >10% for csPCa using the S3M would reduce the number of MRIs and biopsies with 38%, while diagnosing 42% less low-risk PCa at the cost of missing 8% csPCa cases [113]. The strategy of performing only MRI with or without TBx for men with a positive S3M test would save even more biopsies (42%) and low-risk PCa diagnoses (46%), however, at the cost of missing 19% csPCa.

On average, the value of upfront risk stratification with one of the new risk models seems similar to the upfront use of the ERSPC RPCRC to select candidates for MRI. Taking into account the costs and availability of the tests, the ERSPC RPCRC might be preferable. However, to determine the most cost-effective diagnostic pathway in PCa diagnosis, ideally a large prospective cohort study of men biopsied irrespective of risk stratification tool outcome and retrospectively compared performance of all relevant stratification tools should become available for both the initial and repeat biopsy setting.

## 5. Conclusions

There are numerous risk stratification tools available that can help increase the specificity of PSA for the detection of csPCa in the initial and repeat biopsy setting. These tools may thereby refine the PCa diagnostic pathway, improving diagnostic outcome, reducing the burden for patients and making it more cost-effective and acceptable to the general population and health care providers. All risk stratification tools result in a considerable decrease in unnecessary testing and carry a generally small risk of missing csPCa.

Taking into account the costs, RCs using PSA and clinical parameters which perform similarly well as novel, most often more expensive, biomarkers seem to be the preferred choice. However, head-to-head-comparisons of all biomarkers and RCs are necessary. Pre-biopsy prostate MRI has been shown to have more added value in men requiring repeat biopsy than in biopsy-naïve men. Recent studies show evidence for an MRI-directed biopsy management in all men, including biopsy-naïve men.

Merging novel biomarkers, RCs and MRI results in higher diagnostic accuracies and net benefit than the use of these risk stratification tools as standalone test. However, in state-of-the-art clinical decision-making, the patient should benefit from further testing and treatment, even when the diagnostic test is ‘easy-to-perform’. Therefore, the way forward in the current era of prostate MRI is to have an accurate predictive low-cost risk stratification tool. This risk stratification tool as triaging test for the selection of candidates for further testing (e.g. MRI, biopsy) seems to be a multivariable risk assessment based on blood and clinical parameters, potentially extended with information from urine samples, which is free to use, available everywhere, extensively externally validated, and calibrated for different populations. Large prospective and comparative studies remain, however, necessary to fully assess the potentials and risks of these combined strategies.

## Figures and Tables

**Table 1:** Summary table with the performances of currently available risk stratification tools (as standalone tests and merged together) and the diagnostic pathways that combine the tools in the detection of clinically significant prostate cancer (csPCa) (all on average; results can differ between populations).

Risk Stratification Tool	Indication (Biopsy Setting)	Reduced MRIs (%)	Reduced Biopsies (= SBx and/or TBx) (%)	Reduced Low-Risk PCa Diagnoses (= GS 6 or GG 1) (%)	Missed csPCa (≥GS 3 + 4 or ≥GG 2) (%)	Costs ($ or €) *
***Blood-based biomarkers:***						
PHI (cut-off ≥25) --> SBx	Initial and repeat	N/A	40	25	5	$80
4Kscore (cut-off ≥9% csPCa) --> SBx	Initial and repeat	N/A	43	ND	2	$500
***Urine-based biomarkers:***						
PCA3 (cut-off ≥35) --> SBx	Repeat	N/A	67	40	21	$300
PCA3 (cut-off ≥25) plus TMPRSS2-ERG (cut-off ≥10) --> SBx	Initial and repeat	N/A	35	19	10	ND
***Original risk calculators (including PSA and standard clinical data):***						
ERSPC RPCRC (cut-off ≥4% csPCa) --> SBx	Initial and repeat	N/A	32	25	5	Free of charge
PCPT 2.0 (cut-off ≥4% csPCa) --> SBx	Initial and repeat	N/A	16	15	3	Free of charge
Sunnybrook (cut-off ≥4% csPCa) --> SBx	Initial and repeat	N/A	25	22	5	Free of charge
***New risk calculators (including novel biomarkers):***						
4Kscore-ERSPC RPCRC combined (cut-off ≥5% csPCa) --> SBx	Initial	N/A	66	14	2	$500
PCA3-based nomogram Hansen (cut-off ≥30% PCa) --> SBx	Initial	N/A	55	ND	2	$300
MiPS-PCPT RC (cut-off ≥40% PCa) --> SBx	Initial and repeat	N/A	47	10	2	$700
SelectMDx (cut-off ≥-2.8 risk score) --> SBx	Initial and repeat	N/A	42	ND	2	€ 300
S3M (cut-off ≥10% csPCa) --> SBx	Initial	N/A	38	17	6	ND
***Magnetic Resonance Imaging:***						
Upfront MRI + TBx	Initial	0	32	37	4	$1000
After previous negative SBx --> MRI + TBx	Repeat	0	32	38	2	$1000
***Novel biomarkers and MRI merged together:***						
PHI (cut-off ≥35) + MRI suspicion score --> TBx + SBx	Repeat	0	42	13	5	$1080
PHI-density (cut-off ≥0.44) + MRI suspicion score --> TBx + SBx	Repeat	0	35	ND	8	$1080
4Kscore (cut-off <7.5% csPCa) + MRI suspicion score --> TBx + SBx	Initial and repeat	0	15	ND	2	$1500
***Risk calculators including MRI data:***						
MRI-ERSPC RPCRC 3 (cut-off ≥10% csPCa) --> TBx + SBx	Initial	0	14	13	10	$1000
MRI-ERSPC RPCRC 4 (cut-off ≥10% csPCa) --> TBx + SBx	Repeat	0	36	15	4	$1000
Van Leeuwen model (cut-off ≥10% csPCa) --> TBx + SBx	Initial	0	28	13	3	$1000
Truong model (cut-off <70% benign) --> TBx	Repeat	0	29	14	8	$1000
Mehralivand model (cut-off ≥20% csPCa) --> TBx + SBx	Initial and repeat	0	38	ND	11	$1000
***Diagnostic strategies combining tools:***						
Initial 4Kscore (cut-off ≥7.5% csPCa) --> MRI + TBx	Initial and repeat	25	83	75	33	$500–$1500
Initial PCA3 (cut-off ≥35) --> MRI + TBx	Initial	52	76	87	48	$300–$1300
Initial ERSPC RPCRC 3 --> MRI + TBx + SBx	Initial	37	37	23	6	$0–$1000
Initial ERSPC RPCRC 4 --> MRI + TBx	Repeat	37	55	66	17	$0–$1000
Initial SelectMDx (cut-off ≥10% csPCa) --> MRI + TBx + SBx	Initial and repeat	35	35	52	2	€300–€1300
Initial S3M (cut-off ≥10% csPCa) --> MRI + TBx + SBx	Initial and repeat	38	38	42	8	ND

* Including only the estimated costs of the risk stratification tool(s); excluding the costs of biopsy procedures, consultations etc. MRI: magnetic resonance imaging; SBx: systematic biopsy; TBx: MRI-targeted biopsy; PCa: prostate cancer; GS: gleason score; GG: grade group; csPCa: clinically significant prostate cancer; PHI: Prostate Health Index; N/A: not applicable; ND: not determined; 4K: four-kallikrein; PSA: prostate-specific antigen; ERSPC: European Randomized study of Screening for Prostate Cancer; RPCRC: Rotterdam Prostate Cancer Risk Calculator; PCPT: Prostate Cancer Prevention Trial; MiPS: MiProstate Score; S3M: Stockholm-3 model. Red = disadvantage, Orange = neutral, Green = advantage.

## Abbreviations

4K	four-kallikrein
Apps	mobile applications
AUC	area under the curve
bPSA	benign PSA
csPCa	clinically significant prostate cancer
DRE	digital rectal examination
EAU	European Association of Urology
ERSPC	European Randomized study of Screening for Prostate Cancer
fPSA	free PSA
GG	Grade group
GS	Gleason score
hK2	human kallikrein 2
iPSA	intact PSA
ISUP	International Society of Urological Pathology
MiPS	MiProstate Score
MRI	magnetic resonance imaging
mRNA	messenger RNA
NPV	negative predictive value
PCa	prostate cancer
PCA3	prostate cancer antigen 3
PCPT	Prostate Cancer Prevention Trial
PHI	Prostate Health Index
PI-RADS	Prostate Imaging—Reporting and Data System
PLCO	Prostate, Lung, Colorectal, and Ovarian Cancer Screening Trial
PPV	positive predictive value
PSA	prostate-specific antigen
PV	prostate volume
QALYs	quality-adjusted life years
RC	risk calculator
RCT	randomized controlled trial
RPCRC	Rotterdam Prostate Cancer Risk Calculator
S3M	Stockholm-3 model
SBx	systematic biopsy
TBx	targeted biopsy
tPSA	total PSA
TRUS	transrectal ultrasound
USA	United States of America
